# Fe^3+^-Doped TiO_2_ Nanotube Arrays on Ti-Fe Alloys for Enhanced Photoelectrocatalytic Activity

**DOI:** 10.3390/nano6060107

**Published:** 2016-06-06

**Authors:** Jiangdong Yu, Zhi Wu, Cheng Gong, Wang Xiao, Lan Sun, Changjian Lin

**Affiliations:** State Key Laboratory of Physical Chemistry of Solid Surfaces, Department of Chemistry, College of Chemistry and Chemical Engineering, Xiamen University, Xiamen 361005, China; yujiangdong@stu.xmu.edu.cn (J.Y.); boliyusi@163.com (Z.W.); gongcheng@stu.xmu.edu.cn (C.G.); wang.xiaoxm@gmail.com (W.X.); cjlin@xmu.edu.cn (C.L.)

**Keywords:** Fe^3+^ doping, TiO_2_ nanotube arrays, Ti-Fe alloy, anodization, photoelectrocatalytic activity

## Abstract

Highly ordered, vertically oriented Fe^3+^-doped TiO_2_ nanotube arrays (Fe-TNTs) were prepared on Ti-Fe alloy substrates with different Fe contents by the electrochemical anodization method. The as-prepared Fe-TNTs were characterized by scanning electron microscope (SEM), transmission electron microscopy (TEM), X-ray diffraction (XRD), X-ray photoelectron spectroscopy (XPS) and related electrochemical techniques. XPS results demonstrated that Fe^3+^ ions were successfully doped into TiO_2_ nanotubes. The photoelectrochemical activity of Fe-TNTs was compared with that of pure TiO_2_ nanotube arrays (TNTs). The results showed that Fe-TNTs grown on low concentration (0.5 wt %–1 wt % Fe) Ti-Fe alloys possessed higher photocurrent density than TNTs. The Fe-TNTs grown on Ti-Fe alloy containing 0.8 wt % Fe exhibited the highest photoelectrochemical activity and the photoelectrocatalytic degradation rate of methylene blue (MB) aqueous solution was significantly higher than that of TNTs.

## 1. Introduction

Since the discovery of photocatalytic water splitting on the TiO_2_ surface, TiO_2_ has been shown as an admirable photocatalyst to decompose organic contaminant, and it has attracted extensive interest. It is well known that the one-dimensional (1D) materials, including nanotubes, nanofibers, nanowires, and carbon nanotubes, have shown many desirable advantages in photoresponsive properties, and have been reported to improve charge transport in a number of ways [1,2,3,4,5]. Among them, the TiO_2_ nanotube arrays (TNTs), since they were first synthesized by Grimes *et al*. in 2001 [6], have drawn much attention due to their unique properties, such as a highly ordered array structure, a highly specific surface area, outstanding mechanical and chemical stability as well as good charge-transport properties [7]. However, TiO_2_ can only adsorb UV light due to its wide band gap of 3.2 eV (anatase), which limits its efficient use of solar energy since UV light accounts for less than 4% of the solar spectrum. Therefore, many efforts have been explored in order to narrow the band gap and align the band-edge positions, including metal ion doping [8,9], non-metal ion doping [10], noble metal deposition [7,11], semiconductor coupling [12,13], and dye sensitization [14].

Comparatively, doping TiO_2_ with transition metal ions can introduce mid-gap energy levels. Moreover, moderate doping can facilitate the separation of photogenerated electron-hole pairs [15], hence prolonging the lifetime of electron-hole pairs and enhancing the photocatalytic activity [16]. For metal ion doping, Fe^3+^ is considered a promising dopant of TiO_2_ in terms of its ion radius (0.64 Å) close to Ti^4+^ (0.68 Å), and it could replace Ti^4+^ in the lattice without significant alteration of the crystalline structure [17]. Furthermore, Fe^3+^ is relatively stable due to its 3d^5^ (semi-full high spin) electronic configuration, and so the trapped charges can easily release to participate in photoelectrocatalytic reaction [18]. In recent years, it was found that highly ordered TNT layers grown on Ti substrate can be doped with Fe^3+^ using electrochemical anodic oxidation [19] and ultrasound-assisted impregnating-calcination [20], and the photocatalytic activity of TNTs can be significantly enhanced after Fe doping. It is noteworthy that anodization of Ti alloy provides a direct way to incorporate metal ions into the lattice of TiO_2_ [21,22,23]. Mor *et al*. fabricated self-aligned, vertically oriented Ti-Fe-O nanotube arrays by anodic oxidation of Ti-Fe metal films containing different Fe content co-sputtered on fluorine-doped tin oxide (FTO)-coated glass [15]. However, most of Fe elements existed in the form of a-Fe_2_O_3_ and only a small amount of Fe^3+^ was incorporated in the TiO_2_ lattice. In recent years, Fe-doped TNTs have been fabricated by the electrochemical anodization of Ti-Fe alloy in ethylene glycol solution containing 0.25 wt % NH_4_F and 10 wt % H_2_O [24], but were not characterized systematically. In particular, there are no reports on the photoelectrocatalytic activity of Fe-doped TNTs.

In this work, Fe^3+^-doped TiO_2_ nanotube arrays (Fe-TNTs) with different Fe contents were fabricated through electrochemical anodic oxidation using Ti-Fe alloy as the substrate in 0.5 wt % HF aqueous solution and were investigated comprehensively. The photoelectrocatalytic activity of the samples was evaluated by the photoelectrocatalytic degradation of methylene blue (MB) aqueous solution under UV and visible light irradiation. Taking advantage of photocurrent and electrochemical impedance spectroscopy (EIS) techniques, the transfer behavior of photogenerated charges of Fe-TNTs was analyzed.

## 2. Results and Discussion

### 2.1. Morphological Characterization

**Figure 1:**
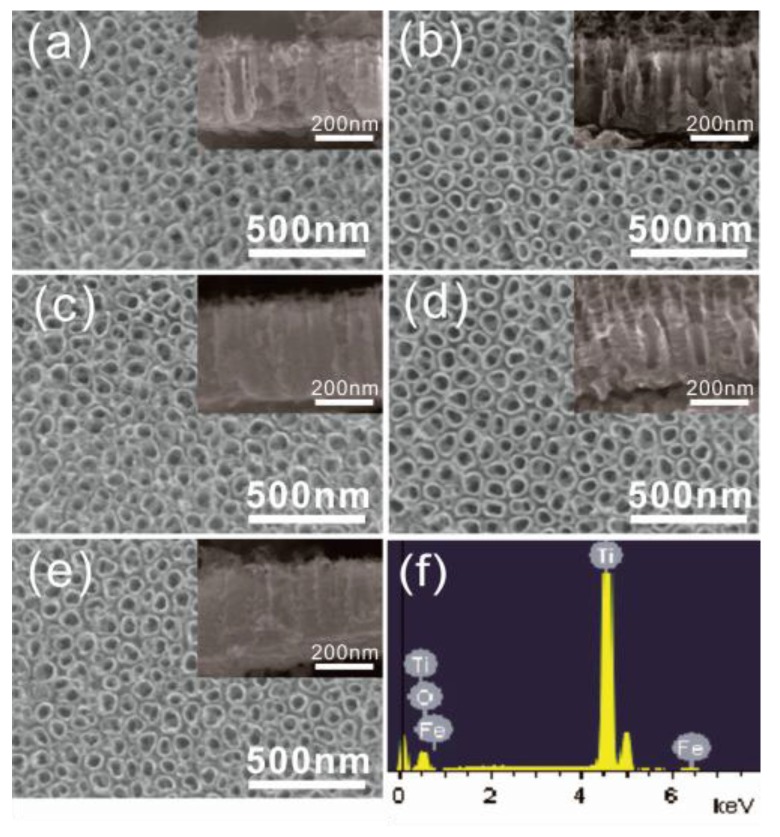
Top-view scanning electron microscope (SEM) images of the nanotube arrays grown on (**a**) Ti; (**b**) Ti05Fe; (**c**) Ti08Fe; (**d**) Ti10Fe; (**e**) Ti50Fe and (**f**) energy dispersive spectrum (EDS) pattern of Ti08Fe. The insets were the corresponding cross-sectional images.

Figure 1a shows the top-view and cross-section-view scanning electron microscope (SEM) images of pure Ti after anodization. Highly ordered and vertically oriented TiO_2_ nanotubes were formed with an average tube diameter of around 60 nm and a tube length of about 250 nm (shown in the inset of Figure 1). Figure 1b–e are the SEM images of Ti-Fe alloys with different Fe content after anodization. It can be seen that the tube diameter and length of the nanotubes grown from Ti-Fe alloy were almost the same as those of TNTs and were independent of the iron content. Nevertheless, the EDS spectrum of the Ti08Fe after anodization is shown in Figure 1f to identify the presence of Fe. The atomic percentage of each element is listed in Table 1. The C element is ascribed to adventitious hydrocarbon from the instrument itself, while the F element results from the anodization electrolyte. Notably, the change of Fe content in Fe-TNTs was consistent with that in Ti-Fe alloys.

### 2.2. Crystalline Structure Characterization

**Figure 2:**
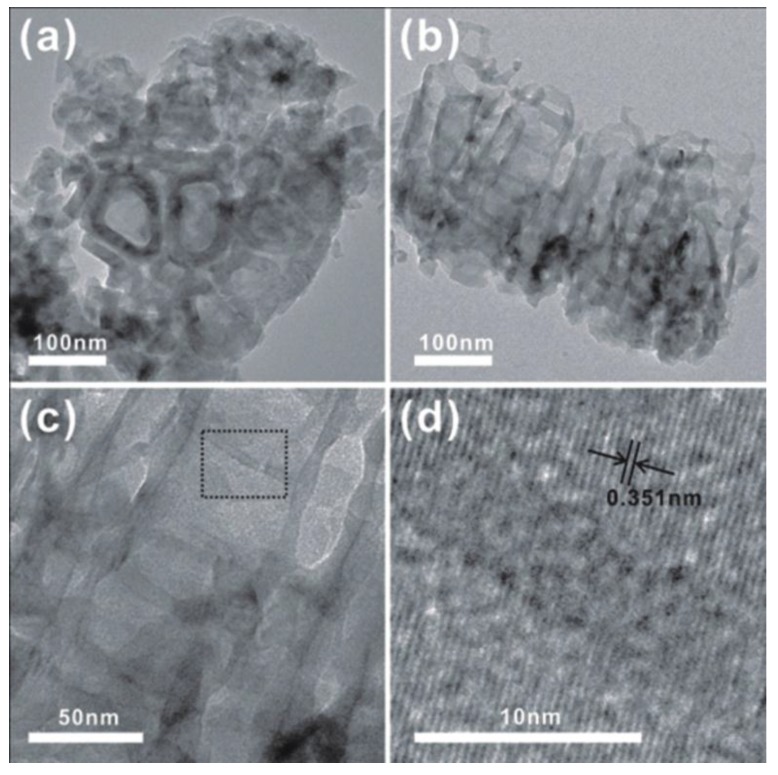
Transmission electron microscopy (TEM) images of Fe-TNTs based on Ti08Fe: (**a**) low magnification of top-view image; (**b**) low magnification of cross-section-view image; (**c**) high magnification of cross-section-view image; and (**d**) HRTEM image. TNTs: TiO_2_ nanotube arrays (TNTs).

Taken as a representative of nanotube arrays, the crystalline structure of the Fe-TNTs prepared by anodizing Ti08Fe alloy was characterized by transmission electron microscopy (TEM). Figure 2a,b show the low-magnification top and cross-sectional images of Ti08Fe, respectively. The nanotubes have a diameter of ~60 nm and a length of ~250 nm, which is consistent with the observation in SEM. The high-magnification cross-sectional image of the sample is shown in Figure 2c. Unlike those Fe-TNTs reported previously [25], the constituent of nanotube array film was homogenous and no iron oxide nanoparticles could be found. Figure 2d displays the corresponding high resolution TEM image. The intact lattice fringe of 0.351 nm corresponded to the interplanar spacing of the (110) plane of anatase TiO_2_, confirming that the Fe-TNTs were anatase.

**Figure 3:**
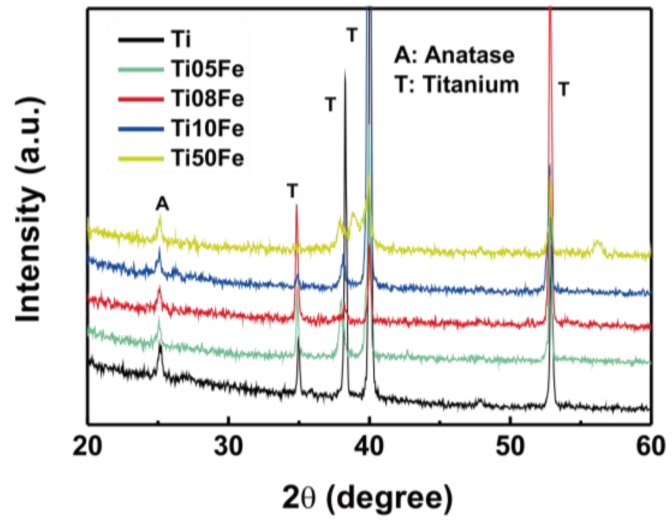
X-ray diffraction (XRD) patterns of the nanotube arrays grown on Tiand different Ti-Fe alloys.

The crystalline structure of samples was further analyzed by X-ray diffraction (XRD). Figure 3 shows the XRD patterns for the anodized Ti-Fe alloys and pure Ti. The predominant peak at approximately 2*q* = 25.2° corresponds to the (101) face of anatase TiO_2_. It is evident that the anatase phase can be identified in all samples after annealing at 450 °C for 30 min. It is noteworthy that the phases of iron oxide still cannot be found, even after the Fe content was further increased to 5.0 wt %. The absence of iron oxide may be attributed to Fe^3+^ diffused along the с-axis and substituted Ti^4+^ in the TiO_2_ lattice [26].

**Figure 4:**
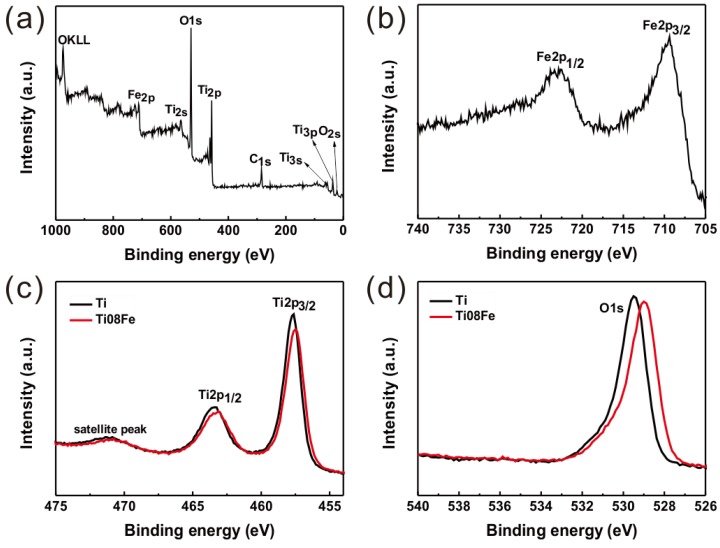
X-ray photoelectron spectroscopy (XPS) survey spectrum of Fe-TNTs (**a**) and high resolution XPS spectra of Fe 2p (**b**); Ti 2p (**c**) and O 1s (**d**) of Fe-TNTs.

To investigate the oxidation state of Fe in Fe-TNTs, the Fe 2p core level was measured by X-ray photoelectron spectroscopy (XPS) and the results are shown in Figure 4b. There were two peaks for as-prepared Fe-TNTs in the high resolution spectrum of Fe 2p. The peaks located at 709.4 and 723.3 eV can be assigned to the Fe 2p_3/2_ and the Fe 2p_1/2_ photoemission spectra [19], respectively, indicating the Fe element mainly exists in the +3 valence state. Meanwhile, Figure 4c,d show the XPS spectra of Ti and O elements of nanotube arrays, respectively. The peak positions of both the Ti 2p core level and the O 1s core level of Fe-TNTs were shifted to a lower binding energy compared to TNTs. This can be attributed to the substitution of Ti^4+^ ions by Fe^3+^ ions, resulting in the formation of Ti-O-Fe bonds [27]. Accordingly, it is further demonstrated that Fe^3+^ ions were doped into the TiO_2_ lattice.

### 2.3. UV-Vis Diffuse Reflectance Spectra

**Figure 5:**
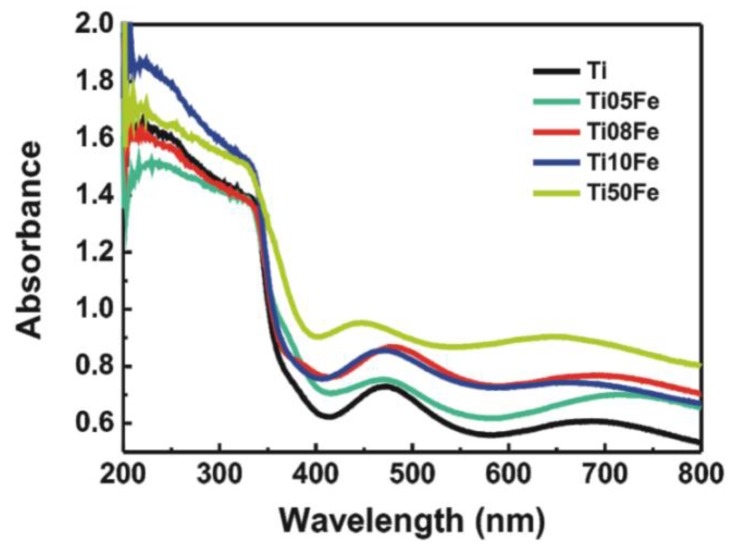
UV-vis diffuse reflectance spectra of TNTs and Fe-TNTs prepared by anodizing Ti-Fe alloys with different Fe content.

Figure 5 shows the UV-vis diffuse reflectance spectra (DRS) of TNTs and Fe-TNTs. All the samples have strong absorption within the ultraviolet light region, corresponding to the band gap of anatase (3.2 eV). Meanwhile, the samples also exhibited several absorption peaks in the range of 400 nm–800 nm, which is attributed to the sub-band-gap states of the TNTs [28]. Compared with the TNTs, the absorption of Fe-TNTs in the visible light region was increasingly improved with the increase of iron content. Notably, the red-shift of the absorption band for Fe-TNTs with the increase of the Fe content was not obvious except for Ti50Fe. The enhanced visible light absorption can be attributed to a sub-band-gap transition between the 3d electron of Fe^3+^ and the TiO_2_ conduction band as well as the d-d transition of Fe^3+^ (2T_2g_ → 2A_2g_,2T_1g_) or the charge transfer transition between interacting iron ions (Fe^3+^ + Fe^3+^ → Fe^2+^ + Fe^4+^) [29,30,31].

### 2.4. Photoelectrochemical and Photoelectrocatalytic Activity

**Figure 6:**
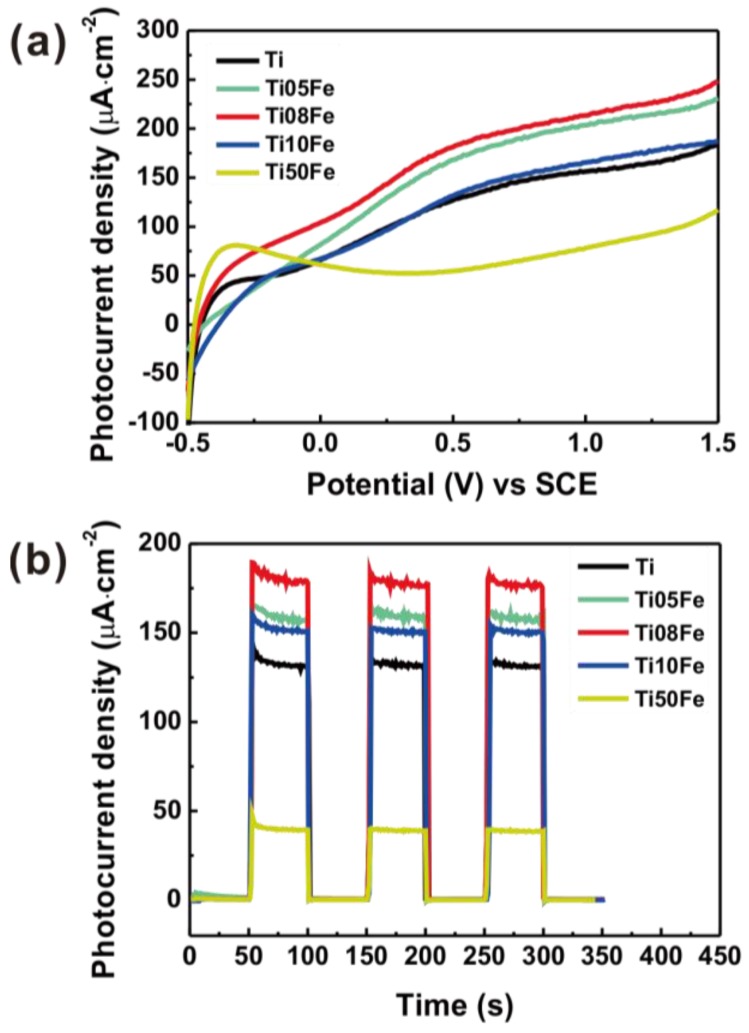
(**a**) *J*-*V* and (**b**) *J*-*t* curves with a bias of 0.6 V for all samples under Xe lamp irradiation.

The performance of Fe-TNTs in photoelectrochemical activity was tested by measuring the photocurrent. Figure 6a shows the photocurrent spectra of TNTs and Fe-TNTs with different Fe content under Xe lamp irradiation (*i.e*., UV and visible light irradiation). The photocurrent densities of all the samples except Ti50Fe increased with the increase of applied potential (*vs*. SCE), indicating the typical photoelectrochemical property of the n-type semiconductor. The photoelectrochemical properties were further examined by transient photocurrent measurement. Figure 6b presents the photocurrent density-time characteristics of the TNTs and Fe-TNTs in 0.1 M Na_2_SO_4_ at an applied bias of 0.6 V (*vs*. SCE) with a pulse of 50 s under UV and visible light irradiation. It can be seen clearly that the photocurrent density increased sharply when the light was switched on, and then decreased to a steady state for each sample. Pure TNTs have a photocurrent density of 131.5 mA·cm^−2^. With the increase of the Fe content, the photoresponse was firstly enhanced but then inhibited. The Ti08Fe sample exhibited the highest photocurrent density of 179.3 mA·cm^−2^, while the Ti50Fe sample had the lowest photocurrent density of 39.8 mA·cm^−2^. Higher photocurrent implies much more enhanced charge separation and a longer lifetime of the photogenerated electron-hole pairs which is closely related to the photoelectrocatalytic activity. When TNTs are irradiated, electrons are excited from the valance band to the conduction band, leaving holes in the valance band. Fe^3+^ dopant with a proper concentration can act as trapping sites, facilitating the separation of electron-hole pairs. However, excessive Fe^3+^ may act as the recombination centers of the photogenerated electrons and holes, which results in the decrease of photocurrent density [32]. Therefore, the doping level of Fe^3+^ plays an important role in the photoelectrochemical behavior of Fe-TNTs and the optimal doping concentration of Fe is 0.8 wt %.

**Figure 7:**
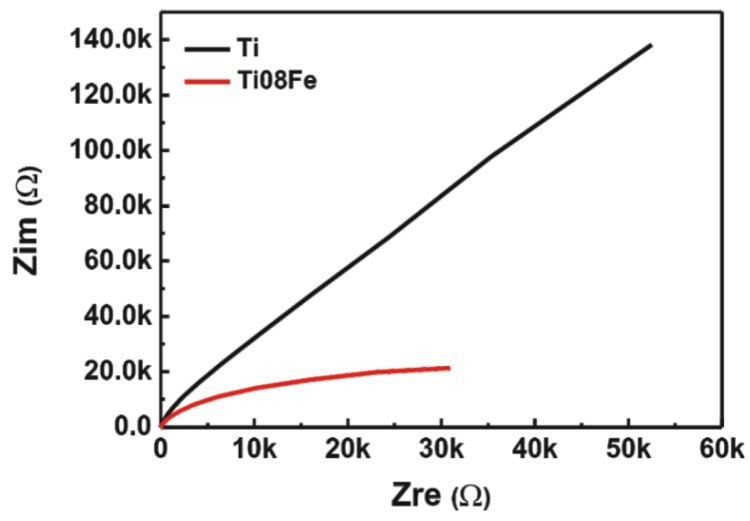
Electrochemical impedance spectroscopy (EIS) Nyquist plots for TNTs and Fe-TNTs prepared by anodizing Ti08Fe alloy under Xe lamp irradiation.

As a widely used electrochemical method, EIS is a powerful and informative tool to study the electron transport processes at the solid-liquid interfaces, and a smaller size of the arc radius on the EIS Nyquist plot means a more rapid rate of electrode reaction [33,34]. Accordingly, the electron transport property of TNTs and Fe-TNTs electrodes was further analyzed by the Nyquist plots of the EIS spectra. As shown in Figure 7, the circular arc radius of Fe-TNTs prepared by anodizing the Ti08Fe alloy was much smaller than that of TNTs under UV and visible light irradiation, suggesting that the introduction of Fe^3+^ ions was beneficial to the separation of the photo-induced carries (e^−^-h^+^) and charge transfer at the solid-liquid interface. Therefore, a higher photoelectrocatalytic rate would be expected by incorporating Fe^3+^ in TNTs.

**Figure 8:**
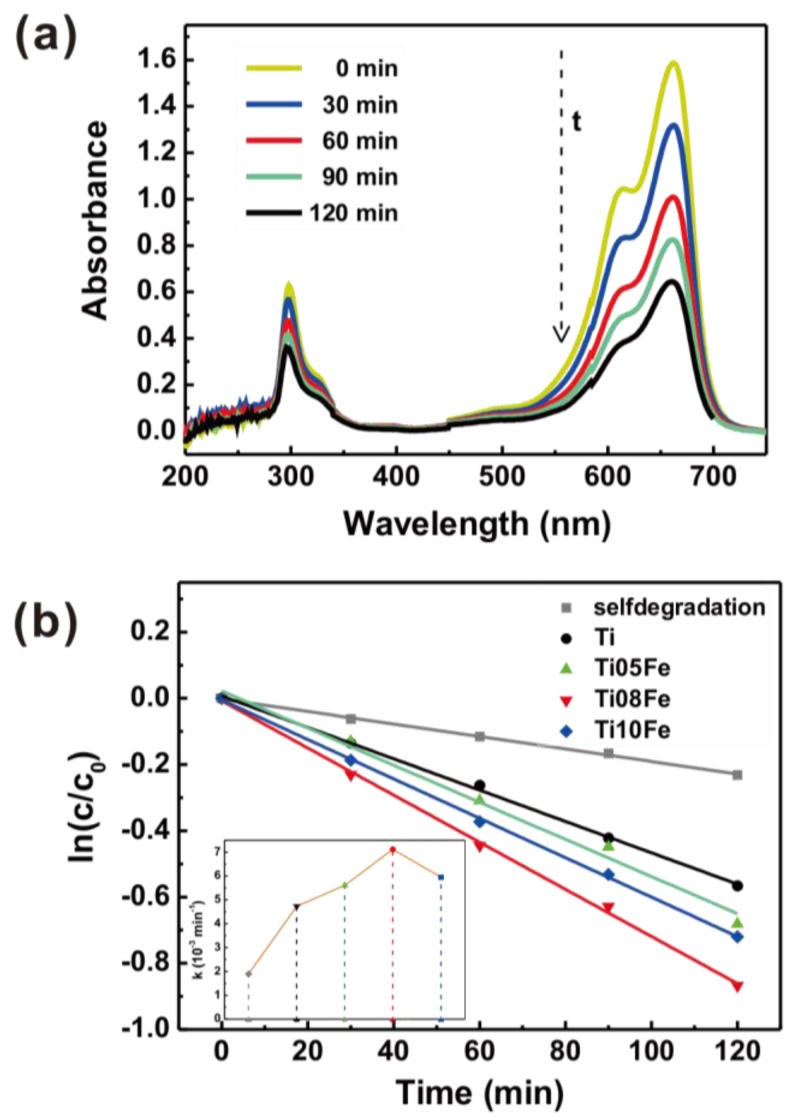
(**a**) UV-vis absorption spectra of methylene blue (MB) solution photoelectrodegraded by Ti08Fe and (**b**) MB degradation kinetic curves of TNTs and Fe-TNTs.

In order to demonstrate the photo-induced application of Fe-TNTs, their photoelectrocatalytic activity was evaluated by degrading MB under UV and visible light irradiation and the applied bias potential was 0.6 V. Figure 8a shows a series of UV-vis absorption spectra of MB solution degraded by Fe-TNTs prepared by anodizing Ti08Fe alloy. It was reported that demethylation could also occur during the process of MB photodegradation, which could be characterized by the blue shift of the absorption peak at 663 nm in the UV-vis absorption spectrum [35]. In Figure 8a, as the irradiation time increased, the absorption peak at 663 nm dropped vertically with no blue shift observed, indicating the MB was effectively photodegraded. Figure 8b shows the photodegradation kinetics of the MB dye over TNTs and Fe-TNTs with different Fe content. The photocatalytic reactions obeyed pseudo-first-order reaction kinetics, which could be expressed by ln(*C*/*C*_0_) = −*kt* with *k* being the apparent first-order reaction constant, while *C*_0_ and *C* are the initial and reaction concentrations of the MB dye, respectively. The reaction constant *k* for TNTs and Ti08Fe was calculated to be 4.73 × 10^−3^ min^−1^ and 7.12 × 10^−3^ min^−1^, respectively. It means that the photoelectrocatalytic activity of Ti08Fe increased 51% more than that of TNTs due to doped Fe^3+^ ions in TNTs.

On the basis of laser flash photolysis measurements [36,37], the photoelectrocatalytic reaction can be described as follows:

TiO_2_ + hν → e_cb_^−^ + h_vb_^+^ (1)

e_cb_^−^ + h_vb_^+^ → hν (2)

h_vb_^+^ + Red → Red^+^ (3)

e_cb_^−^ + O*_x_* → O*_x_*^−^ (4)

Ti^4+^ + e_cb_^−^ → Ti^3+^ (5)

OH^−^ + h_vb_^+^ → OH^•^ (6)

Ti^3+^ + OH^•^ → Ti^4+^ + OH^−^ (7)

OH^•^ + Red → Red^+^ (8)

Ti^3+^ + O*_x_* → O*_x_*^−^ (9)

**Figure 9:**
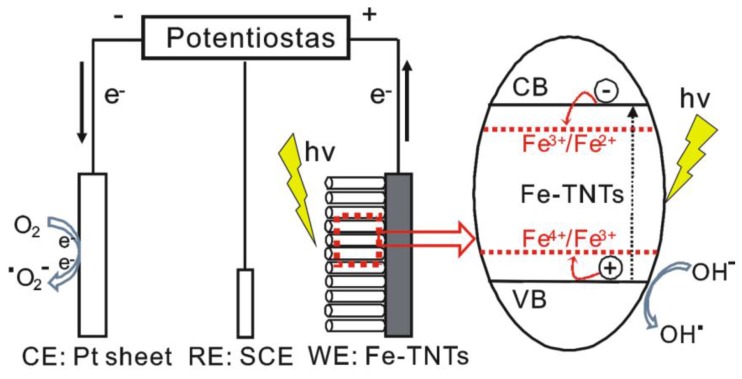
Schematic illustrating the separation and transport of charge carriers in the process of photoelectrocatalytic degradation over Fe-TNTs.

Upon UV and visible light irradiation, the TiO_2_ could be excited to generate electron-hole pairs (Equation (1)), while the photogenerated electron-hole pairs could easily recombine (Equation (2)). The separated holes and electrons induced an oxidation (Equation (3)) and reduction (Equation (4)) reaction, respectively. At the same time, a series of thermal reactions and catalytic reactions also happened, such as those where trapping electrons or holes could produce Ti^3+^ or other active groups (Equations (5) and (6)), respectively. Then the Ti^3+^ and other active groups would take part in the subsequent redox reactions (Equations (7)–(9)). Most of these electrons and holes recombine within the first few tens of picoseconds after the photoexcitation event [36], suggesting a low quantum efficiency in pure TiO_2_. Figure 9 illustrates the main charge-transfer behavior in Fe-TNTs. After doping the TNTs with Fe^3+^, Fe^3+^ can trap photogenerated holes (Equation (10)) due to the energy level for Fe^4+^/Fe^3+^ above the valence band edge of anatase TiO_2_. The trapped photogenerated holes in Fe^4+^ can migrate to the surface and absorb hydroxyl ions to produce hydroxyl radicals. At the same time, Fe^3+^ can also trap photogenerated electrons (Equation (12)) due to the energy level for Fe^3+^/Fe^2+^ below the conduction band edge of anatase TiO_2_ [38,39,40,41]. Subsequently, Fe^2+^ could be oxidized to Fe^3+^ by transferring electrons to absorbed O_2_ on the surface of TiO_2_ (Equation (13)).

Fe^3+^ + h_vb_^+^ → Fe^4+^ (10)

Fe^4+^ + OH^−^ (ads) → Fe^3+^ + OH^•^ (11)

Fe^3+^ + e_cb_^−^ → Fe^2+^ (12)

Fe^2+^ + O_2_(ads) → Fe^3+^ + O_2_^−^ (13)

Accordingly, the doping of Fe^3+^ could depress the recombination of electron-hole pairs and prolong the lifetime of the carriers, which is beneficial for improving the quantum efficiency of the photoelectrocatalytic reaction. When a bias potential was applied, the photogenerated electrons that collected at the photoanode could be transferred to the Pt sheet through the external circuit. The separation of the photogenerated electron-hole pairs was efficiently promoted and thus the photocatalytic efficiency was improved remarkably.

## 3. Experimental Section

### 3.1. Preparation of Fe-TNTs

Pure Ti and Ti-Fe alloys with different Fe content (containing 0.5 wt %, 0.8 wt %, 1.0 wt %, and 5.0 wt %, and namely Ti05Fe, Ti08Fe, Ti10Fe and Ti50Fe) were prepared by using an arc-melting apparatus. TNTs and Fe-TNTs were prepared by electrochemical anodization of Ti substrate and Ti-Fe substrates, respectively. Prior to the anodization, the samples were cut into pieces (10 mm 10 mm 2 mm) and polished to a mirror finish, sequentially followed by ultrasonically cleaned in acetone, alcohol, and distilled water. The anodization was performed in 0.5 wt % HF aqueous solution for 20 min at 15 V in a two-electrode cell with samples as the working electrode and platinum foil as the counter electrode at room temperature. After anodization, samples were immediately rinsed with deionized water and dried in air. In order to convert the amorphous phase to the crystalline form, samples were annealed at 450 °C in air for 30 min with a heating rate of 5 °C·min^−1^ and a cooling rate of 2 °C·min^−1^.

### 3.2. Characterization of Fe-TNTs

The morphologies of TNTs and Fe-TNTs were studied by using a field-emission scanning electron microscope (FE-SEM, Hitachi S4800, Hitachi, Tokyo, Japan) and a transmission electron microscopy (TEM, JEM 2100, JEOL, Tokyo, Japan). Additionally, a selected area energy dispersive spectrum (EDS) was performed on the Hitachi-S4800 SEM. The structure characterization of all samples was conducted by X-ray diffraction (XRD, Philips, Amsterdam, The Netherlands, PanalyticalX’pert, Cu Karadiation (λ = 1.5417 Å)), operated at 40 kV and 30 mA. The composition of samples was analyzed by X-ray photoelectron spectroscopy (XPS, VG, Physical Electrons Quantum 2000 Scanning EscaMicroprob, Al Kα radiation, Physical Electronics, Inc., Chanhassen, MN, USA). The binding energies were normalized to the signal for adventitious carbon at 284.8 eV. UV-vis diffuse reflectance spectra (DRS) of the samples were carried out by a UV-vis-NIR spectrophotometer (Varian Cary 5000, Agilent, Santa Clara, CA, USA).

### 3.3. Photoelectrochemical and Electrochemical Measurement

All the measurements were carried out in a standard three-electrode configuration with a supporting electrolyte of 0.1 M Na_2_SO_4_ aqueous solution using the samples, Pt wire and a saturated calomel electrode (SCE) as the working electrode, counter electrode and reference electrode, respectively. Photocurrent was recorded using the Ivium EC portable analyzer (Ivium Technologies BV, Eindhoven, The Netherlands). Additionally, a 300 W Xe lamp was employed as the light source to keep an illumination intensity of 100 mW·cm^−2^. The EIS spectra were measured by applying an AC voltage of 10 mV amplitude within the frequency range of 10^5^–10^−2^ Hz.

### 3.4. Photoelectrocatalytic Measurement

**Figure 10:**
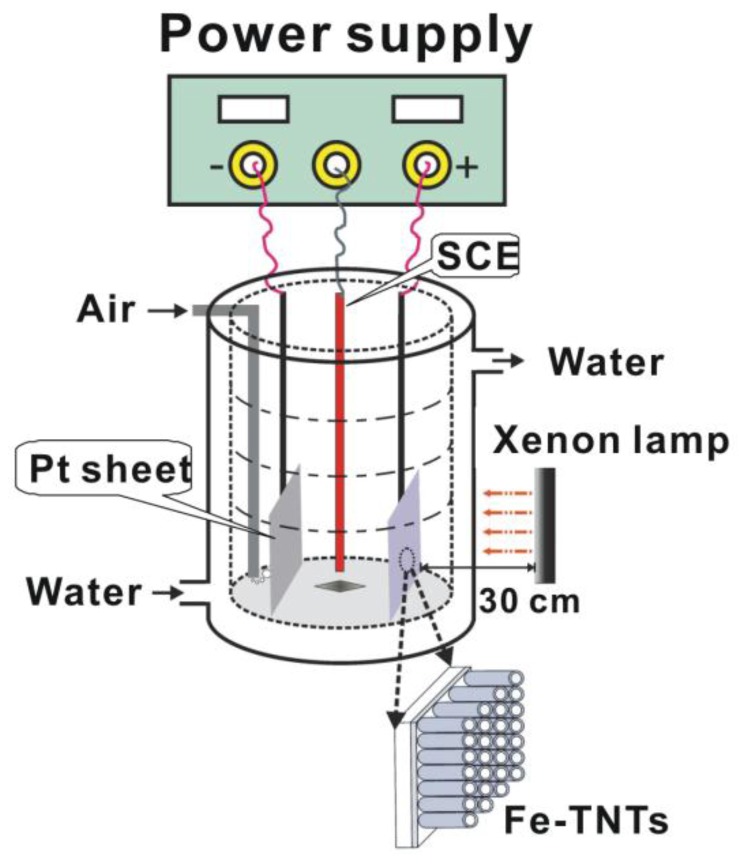
Schematic diagram of a photoelectrochemical cell for photoelectrocatalytic degradation of organic pollutant by capitalizing on Fe-TNTs (or TNTs) as working electrode, Pt as counter electrode, and saturated calomel electrode (SCE) as the reference electrode, respectively.

The photoelectrocatalytic activity of the samples was investigated by the degradation of a MB aqueous solution with an initial concentration of 10 mg·L^−1^ as model pollutant and 0.1 M Na_2_SO_4_ as supporting electrolyte in a self-building quartz glass reactor with a water jacket to control the reaction temperature, as shown in Figure 10. A 300 W Xe lamp was employed as the light source. Prior to the photoelectrocatalytic degradation, the samples were soaked in 10 mg·L^−1^ MB aqueous solution for 30 min while bubbling with air to reach adsorption equilibrium. After irradiation started, the solution periodically taken from the reactor was analyzed with a UV-vis spectrophotometer (Unico UV-2102 PC, Unico Instrument Co., Ltd., Shanghai, China).

## 4. Conclusions

Highly ordered Fe-TNTs were successfully fabricated by direct anodization of Ti-Fe alloys with different Fe contents. In all cases, ordered nanotube array layers homogeneously grew on Ti and Ti-Fe alloys, and no appreciable structure and morphology difference could be observed. The Fe^3+^ doping at a relatively weak level can obviously enhance the photoelectrochemical activity of TNTs. On the contrary, when the Fe^3+^ doping amount exceeded a certain level, the photoelectrochemical activity of TNTs remarkably decreased. The Fe-TNTs prepared by anodizing Ti08Fe alloy exhibited the highest photocurrent density and photoelectrocatalytic degradation rate of MB. The Fe-TNTs prepared by anodizing the Ti-Fe alloys may be a promising material not only for organic pollutant degradation but also for the other photocatalytic applications due to abundant Ti and Fe in the earth.

## Figures and Tables

**Table 1:** Composition of the as-prepared Fe-TNTs (TiO_2_ nanotube arrays) based on different Fe-Ti alloys (atomic percentage according to energy dispersive spectrum (EDS)).

Samples	C	F	Ti	O	Fe
Ti05Fe	2.66	2.52	42.65	51.61	0.55
Ti08Fe	3.13	2.14	42.19	51.67	0.87
Ti10Fe	3.20	2.60	41.66	51.43	1.11
Ti50Fe	3.04	0.81	38.61	52.70	4.83
